# An Unusual Case of an Acute Mesenteroaxial Gastric Volvulus Secondary to a Hiatal Hernia

**DOI:** 10.7759/cureus.31296

**Published:** 2022-11-09

**Authors:** Sukhjinder Chauhan, Rasiq Zackria, John K Ryan

**Affiliations:** 1 Internal Medicine, Mountainview Hospital, Las Vegas, USA; 2 Gastroenterology and Hepatology, Sunrise Health GME (Graduate Medical Education) Consortium, Las Vegas, USA; 3 Gastroenterology and Hepatology, Comprehensive Digestive Institute of Nevada, Las Vegas, USA

**Keywords:** gastric outlet obstruction, gastropexy, mesenteroaxial volvulus, hiatal hernia, gastric volvulus

## Abstract

Gastric volvulus is characterized by the abnormal twisting of the stomach along its axis. It is a rare condition that can develop secondary to an underlying gastrointestinal anatomic defect such as a hiatal hernia. Gastric volvulus may present acutely with symptoms of gastric outlet obstruction and can lead to potentially fatal complications, if not treated in a timely manner. We present the case of a 74-year-old woman who presented with an acute mesenteroaxial gastric volvulus with gastric outlet obstruction that developed secondary to a large hiatal hernia.

## Introduction

Gastric volvulus refers to the abnormal rotation of the stomach, usually greater than 180 degrees, along its vertical or horizontal axis. It was first described by Berti in 1866 and was treated surgically for the first time by Berg in 1897 [1,2]. About 80%-90% of the affected individuals are adults, and the incidence typically peaks after the fifth decade of life [3,4]. It is a rare clinical entity with only a total of 90 cases reported between 1999 and 2022 [5]. This report describes a case of an acute mesenteroaxial gastric volvulus secondary to an untreated hiatal hernia.

## Case presentation

A 74-year-old Caucasian woman presented to our hospital with a four-day history of nausea and vomiting that later progressed to hematemesis. Her symptoms began four days ago after eating a large meal at a restaurant. The vomitus consisted mainly of food particles. However, one day prior to admission, she experienced several episodes of hematemesis. She denied any history of melena, hematochezia, fever, or chills.

The patient had a history of hypertension, hyperlipidemia, type II diabetes, transient ischemic attack, depression, chronic back pain, ulcerative colitis in remission, gastroesophageal reflux disease (GERD), peptic ulcer disease, and hiatal hernia. She was diagnosed with a hiatal hernia of unknown type/size in 2017 after evaluation of recurrent GERD symptoms and was suggested to pursue fundoplication. However, she was lost to follow-up. Her past surgical history was significant for a total abdominal hysterectomy. There was no history of any other abdominal surgeries. Her medications included lisinopril, hydrochlorothiazide, metolazone, omeprazole, calcium carbonate, pregabalin, tramadol, duloxetine, and as-needed acetaminophen.

**Figure 1:**
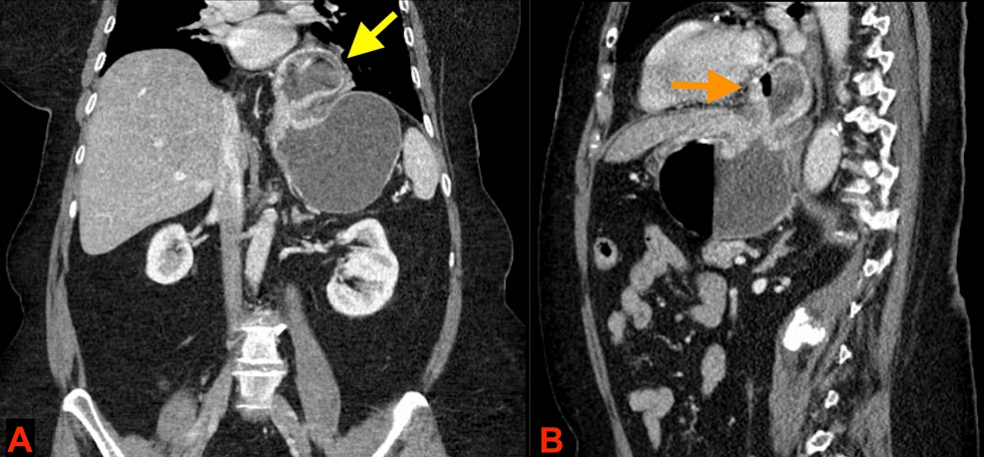
CT abdomen and pelvis with IV contrast demonstrating a large hiatal hernia with a suspected mesenteroaxial gastric volvulus. A: Coronal view, and B: sagittal view. Yellow arrow in image A shows gastric body in the hiatus. Orange arrow in image B shows gastric body located in the posterior mediastinum.

On admission, the patient was afebrile and hemodynamically stable. Abdominal examination revealed a distended abdomen that was tender to deep palpation. Bowel sounds were mildly diminished, and no guarding or rebound tenderness was noted. Complete blood count was remarkable for a leukocyte count of 15.4 x 10^9^/L, a hemoglobin level of 16.1 g/dL, and a hematocrit of 48.4%. A comprehensive metabolic panel revealed blood urea nitrogen of 20 mg/dL, creatinine of 1.18 mg/dL (baseline creatinine: 0.80 mg/dL), and venous lactate of 1.0 mg/dL. Liver function tests were normal. Urinalysis was remarkable for bacteriuria. Chest X-ray revealed a moderate-sized paraesophageal hiatal hernia along with mild bibasilar atelectasis that was more prominent on the left side. Computed tomography (CT) of the abdomen and pelvis (Figures 1A, 1B) demonstrated a hiatal hernia and a suspected mesenteroaxial gastric volvulus with fluid distention of the stomach.

On admission, the patient received intravenous fluids, ondansetron, and pantoprazole. The gastroenterology team recommended urgent esophagogastroduodenoscopy (EGD). During endoscopy, retroflexion within the stomach revealed a large hiatal hernia with a portion of the antrum trapped above the diaphragm; the type of hiatal hernia could not be ascertained as there was evidence of complete gastric outlet obstruction (GOO) secondary to gastric volvulus. Given these endoscopic findings, emergency evaluation by surgery was recommended and an exploratory laparotomy by the surgery team was performed with the finding of hiatal hernia with intrathoracic portion of the stomach causing gastric volvulus. Reduction of the volvulus and hiatal hernia was done. A gastrostomy tube was placed, and gastropexy was also performed to prevent future gastric dilatation and volvulus.

## Discussion

**Figure 2:**
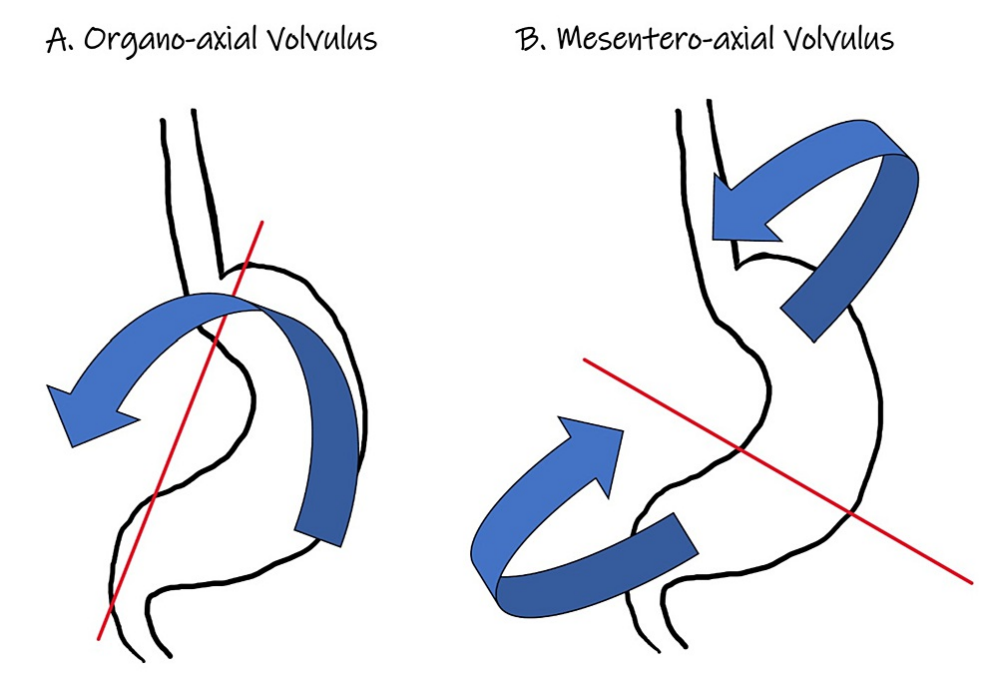
Illustration made by author RZ demonstrating the two different types of gastric volvulus classified based on the axis of stomach rotation. A: Organo-axial type twists along the line connecting the cardia and pylorus along the luminal axis of the stomach. It is the most common type and is usually associated with diaphragmatic and vascular compromise. B: Mesenteroaxial type rotates around a perpendicular plane to the luminal axis from the lesser to greater curvature. Diaphragmatic defects are less common; however, chronic symptoms can be present.

Depending on the etiology, gastric volvulus can be classified as primary or secondary. Primary gastric volvulus develops due to abnormalities of the gastric ligaments, which can result in failure of gastric fixation. Secondary gastric volvulus is more common and develops due to anatomical defects of the phrenoesophageal ligament resulting in the development of a hiatal hernia. Paraesophageal hernia is the most common cause of secondary gastric volvulus in adults [6]. Another method to classify gastric volvulus is based on the axis of stomach rotation as depicted in Figure 2. The most common type of abnormal rotation is organo-axial volvulus (Figure 2A), which is characterized by gastric rotation along the longitudinal cardiopyloric axis usually drawn from the gastroesophageal junction (GEJ) to the pylorus. Mesenteroaxial volvulus (Figure 2B) occurs when the gastric rotation is perpendicular (90 degrees) to the short transverse axis and the pylorus rotates above the GEJ. Mesenteroaxial volvulus is not commonly associated with a hiatal hernia. Occasionally, a combination of these two types may occur [7,8].

The presentation may be acute or chronic. Acute gastric volvulus is a surgical emergency and presents with symptoms of GOO including acute epigastric abdominal pain, nausea, vomiting, and inability to tolerate oral intake. The Borchardt’s triad of upper abdominal or chest pain, severe retching, and an inability to pass a nasogastric tube may be seen in up to 70% of acute cases, primarily in organo-axial volvulus. Mucosal sloughing due to ischemia or mucosal tear secondary to retching can occur, resulting in hematemesis. Chronic gastric volvulus typically presents with nonspecific symptoms, such as epigastric discomfort, dysphagia, early satiety, heartburn, and abdominal bloating [6,8].

Imaging studies, including plain radiography, fluoroscopy, and CT, are used to establish the diagnosis. Plain abdominal radiographs usually reveal a single gas bubble with a paucity of air in the distal bowel. CT abdomen is considered the gold-standard imaging modality; it can be used to confirm gastric rotation and obstruction and can aid in locating a transitional point [9,10]. EGD can be both diagnostic and therapeutic; however, diagnosis via endoscopy may be established in only 28%-45% of cases [10]. Distortion of the gastric anatomy and difficulty in advancing the gastroscope through the stomach or pylorus are highly suggestive of gastric volvulus with GOO [10-12].

The initial management of an acute gastric volvulus consists of fluid resuscitation, electrolyte correction, and gastric decompression by placing a nasogastric tube. Primary surgical management involves reduction and derotation of the volvulus, repair of underlying anatomic defects, and/or gastropexy with gastrostomy tube placement. Gastropexy with one or two gastrostomy tubes prevents recurrence by affixing the stomach to the anterior abdominal wall; in addition, this provides postoperative decompression and allows access to enteral nutrition [10,13]. Endoscopic derotation and gastric fixation may be pursued in patients with high surgical risk; however, this does not address the associated anatomic abnormalities and is not ideal for patients with a secondary volvulus [14,15]. Failure to promptly recognize a gastric volvulus can result in life-threatening complications, including gastric ischemia, necrosis, and perforation, requiring partial or total gastrectomy [16].

Our patient presented with epigastric pain, nausea, and vomiting had a hiatal hernia, and the gastroscope was unable to be advanced from the stomach into the duodenum during endoscopy, all of which were consistent with the Borchardt's triad suggestive of acute gastric volvulus. CT scan confirmed the presence of an acute mesenteroaxial gastric volvulus. The condition was managed promptly via surgical reduction of the volvulus and gastropexy with gastrostomy tube placement.

## Conclusions

Gastric volvulus is a rare condition that can develop secondary to an anatomic defect, such as a hiatal hernia, and may present as an abdominal emergency. The diagnosis can be confirmed via imaging and is typically corrected surgically. Delayed diagnosis and management can lead to the development of life-threatening complications. Therefore, it is crucial to maintain a high index of clinical suspicion in patients who present with symptoms of GOO in the setting of gastrointestinal anatomic abnormalities.
